# Protocol for MInimizing the Risk of Metachronous Adenomas of the CoLorectum with Green Tea Extract (MIRACLE): a randomised controlled trial of green tea extract versus placebo for nutriprevention of metachronous colon adenomas in the elderly population

**DOI:** 10.1186/1471-2407-11-360

**Published:** 2011-08-18

**Authors:** Julia C Stingl, Thomas Ettrich, Rainer Muche, Martina Wiedom, Jürgen Brockmöller, Angela Seeringer, Thomas Seufferlein

**Affiliations:** 1 Institute of Pharmacology of Natural Products and Clinical Pharmacology, University Ulm, Germany; 2 Department of Internal Medicine I, University Hospital, Martin-Luther-University Halle-Wittenberg, Germany; 3 Institute of Epidemiology and Medical Biometry, University Ulm, Germany; 4 Department of Clinical Pharmacology, University Göttingen, Germany

## Abstract

**Background:**

Prevention of colorectal cancer is a major health care issue. People who have undergone colonoscopy screening and had colorectal polyps removed have a higher risk of being diagnosed with polyps again compared to the normal population. Therefore, it would be ideal to find appropriate means that effectively help to prevent the reoccurrence of polyps after polypectomy. So far, pharmaceutical chemoprevention with NSAIDs including aspirin has been shown to be effective but not gained general acceptance due to side effects. Nutraceuticals such as polyphenols from tea plants have demonstrated remarkable therapeutic and preventive effects in molecular, epidemiological and clinical trials. However, placebo-controlled trials demonstrating the efficacy of nutraceuticals for the (secondary) prevention of colorectal polyps as precursors for colorectal cancer are missing.

**Methods/Design:**

We present the design of a randomized, placebo controlled, multicentre trial to investigate the effect of diet supplementation with green tea extract containing 300 mg epigallocatechin gallate (EGCG), the major polyphenol in green tea, on the recurrence of colon adenomas. Patients who have undergone polypectomy for colonic polyps will be randomized to receive either green tea extract containing 150 mg EGCG two times daily or a placebo over the course of three years. After a one month run-in period in which all patients will receive the active intervention, 2534 patients will be randomized, and 2028 patients are expected to complete the whole study course. Incidence, number and histology of adenoma at endpoint colonoscopy at three years will be compared in both groups.

**Discussion:**

The beneficial safety profile of decaffeinated green tea extract, the quantifiable and known active content EGCG, and the accumulating evidence of its cancer preventive potential require, in our view, a validation of this compound for the nutriprevention of colorectal adenoma. Good accessibility and low costs might render this neutraceutical a top candidate for wider use as food supplement in colon cancer prevention.

**Trial registration:**

ClinicalTrials.gov: NCT01360320

## Background

The use of nutritional compounds for disease prevention and health maintenance is an emerging field, since nutritional products are usually less afflicted with the risk of severe side effects, and long-term experience in humans exists. Especially for cancer prevention, associations between diet and lifestyle have been established [1].

Green tea belongs to the oldest drinks of mankind, and its high levels of bioactive polyphenols provide the potential for therapeutic and preventive application. Supplementation of diet with green tea polyphenols as a nutraceutical has already been studied in the context of cancer, diabetes and cardiovascular disease [2-7].

About 30% of the content of green tea is polyphenols. The monomer flavan-3-ols are called catechins, and contribute to the astringent/bitter taste of green tea. Among all catechins in green tea the most important are 59% (-)-epigallocatechin-3-gallate (EGCG), 19% (-)-epigallocatechin (EGC), 13.6% (-)-Epicatechin-3-gallate (ECG), and 6.4% (-)-epicatechin (EC) [8].

Due to its presumed beneficial effects on cancer initiation and early cancer progression green tea polyphenols might be especially suited for cancer prevention rather than for cancer treatment [9]. The majority of colorectal cancers, the most frequent cancer type in men, shows a slow progression from normal mucosa to adenoma or advanced lesion (adenoma-carcinoma sequence), therefore allowing efforts for prevention of colorectal cancer [10]. Several studies on the chemoprevention of colorectal cancer have been performed using compounds such as acetyl salicylic acid (ASS) or cyclooxygenase (COX) isoenzyme 2-specific nonsteroidal anti-inflammatory agents (NSAIDs) [11-17]. NSAIDs seem to have a beneficial effect in preventing the development and/or recurrence of adenomas. However, the benefit risk ratio of daily use of these agents over years does not seem to justify a broad use for primary or secondary chemoprevention due to adverse effects such as gastrointestinal hemorrhage, peptic ulcers and potential cardiovascular toxicity [18]. Therefore, COX inhibitors have been accepted as chemopreventive agents only for patients with a very high risk of developing colorectal cancer, such as those with familial adenomatous polyposis [19].

Green tea extract and its major active component EGCG have been studied in several models for their preventive properties on the development of colon adenomas. In the Apc^min ^mouse, a model for colon adenoma development, a tremendous effect on the number and growth of adenomas was detected upon feeding the animals with EGCG-supplemented drinking water [20,21].

In addition, EGCG even inhibits the growth of established human colorectal cancer cells and induces apoptosis, which is accompanied by inhibition of COX-2 expression and reduced activation of the epidermal growth factor receptor tyrosine kinase family [22,23].

One small pilot study on the supplementation of green tea consumption with capsules containing green tea extract in Japanese patients with a history of colon adenomas resulted in a 50% reduction of the incidence of metachronous adenomas in the supplementation group compared to the placebo group after one year [9]. Epidemiological studies in Asian countries also point to a preventive effect on colon cancer from high daily intake of green tea (more than 5 cups daily) [24,25].

Since green tea extract - in contrast to green tea itself - is decaffeinated, diet supplementation with EGCG from green tea extract may constitute a nutriprevention with an extremely favorable safety profile. We report here on the design of a large, placebo-controlled prospective trial on the effect of diet supplementation with EGCG on colorectal adenoma prevention.

## Methods/Design

### Design and setting

The trial follows a prospective randomized controlled design comparing the twice daily intake of green tea extract containing 150 mg EGCG supplement to a placebo. Patients will be randomized after a verum run-in period of one month to receive either green tea extract containing 150 mg EGCG two times daily or placebo over the course of three years.

### Study outcomes

#### Primary outcome

The primary outcome parameter will be the incidence of any (n equal to or larger than 1) metachronous colorectal adenomas (tubulovillous, tubular, villous and serrated lesions) at the 3 year follow-up colonoscopy in both groups.

#### Secondary outcomes

The occurrences, number, localization, size and histological subtypes of adenomas or mucosal lesions and invasive growth, as well as the time to detection of new colorectal carcinoma (assuming that in several patients more than one colonoscopy will be performed within 3 years) and the frequency of carcinoma, will be assessed as secondary outcome parameters in both groups. In addition, genetic and biochemical biomarkers for the recurrence of adenoma or the development of dysplasia and carcinoma will be analyzed in the blood samples entered into the biobanking subproject. Paraffin-embedded tissue samples will be collected in participants who show a high grade dysplastic adenoma or serrated adenoma at the follow-up colonoscopy. In these samples, besides the histological analyses the possibly therapeutically relevant tumor-genetical alterations including K-ras mutation, B-raf mutation or specific microRNA will be analyzed.

In an independent project, the nutrikinetics and nutrigenetics of EGCG and other green tea catechins will be assessed in healthy volunteers with the capsules used in this study.

### Sample size calculation

The sample size estimation was derived from the existing chemopreventive studies reporting a re-incidence of metachronous adenomas of about 45% at follow-up colonoscopy [13]. Assuming an effect similar to that observed for ASS, chemoprevention might lead to a relative risk reduction of 12% [13]. Therefore, if ECGC intake leads to a similar relative risk reduction of 11% and if there is a recurrence of adenomas in 45% of the patients in the placebo group, 40% of the patients taking green tea extract should have a recurrent adenoma after three years. Sample size estimation was performed using the Mantel-Haenszel test stratifying for the use of low-dose ASS, assuming that 20% of the study participants will be on low-dose (≤ 100 mg) ASS due to the frequent use of low-dose ASS in the age group of the participants (50 years and above). Assuming a recurrence rate of 38.3% in the combined verum green tea and low-dose ASS arm, and of 47.1% in the placebo/no ASS group, a sample size of n = 1203 (both in verum and placebo) is required for the intention to treat (ITT) sample with significance level of 5% and a power of 80% (stratified one-sided Mantel-Haenszel test, NQUERY 7.0 software, Statistical Solutions, Cork, Ireland).

Assuming a drop-out rate of 5% of patients for the control colonoscopy, 1267 randomized patients per arm will be required, resulting in randomization of 2534 patients after the run-in period.

A drop-out rate of 20% is assumed for regular capsule intake. Thus, 1014 patients will be evaluable for the per-protocol analysis of the green tea effect. For exploratory analysis of potential confounders, this sample size is sufficient to include 30-40 variables inclusive dummy-coding with around 20 events per variable into the model.

### Participants

The study will be performed nationwide in Germany. Patients will be recruited from gastroenterological centers that are performing a high number of screening and interventional colonoscopies. Participants undergoing screening colonoscopy who are diagnosed with colorectal adenomas will be randomized within 6 months after polypectomy if they give informed consent. Participants who are willing to participate in the trial will undergo a short screening interview and then enter the run-in phase of 4 weeks, in which all participants of the trial will receive verum. This run in-phase is designed to obtain information about potential and unforeseen side effects of green tea extract intake that might affect the well-being of the participants or the blinding of the trial. Randomization will follow after a successful run-in period. Participants who are not compliant to the study protocol or do not tolerate the green tea extract supplementation will not be randomized.

**Figure 1:**
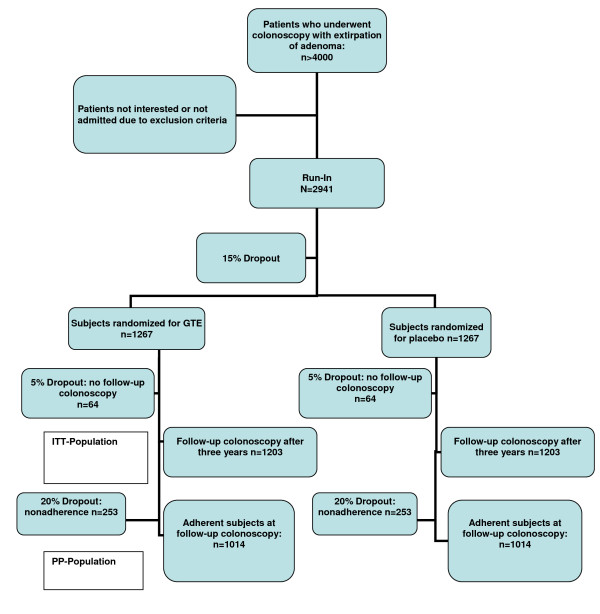
**Flow Diagram of the progress through the phases of the study**. Assuming a drop-out rate of 5% of patients for the control colonoscopy, 1267 randomized patients per arm will be required, resulting in randomization of 2534 patients after the run-in period. 1203 patients per arm are expected to be available for the ITT analysis undergoing control colonoscopy after three years. A drop-out rate of 20% is assumed for regular capsule intake. Thus, 1014 patients will be evaluable for the per-protocol analysis of the green tea effect.

As judged from similar trials, we expect a 15% dropout rate after run-in. Thus, 2941 participants will be included in the run-in phase in order to achieve a total number of 2534 randomized participants (Figure 1).

In Germany, about 500,000 individuals undergo screening colonoscopies each year. A large colonoscopy center typically performs more than 3000 colonoscopies per year. In 10-15% of these screenings, adenomas are detected and removed. We expect that about half of these cases will be eligible for study inclusion. About 30 colonoscopy centers will take part in this study, in order to allowing us to recruit the planned number of participants in the intended 3-year period.

### Inclusion & exclusion criteria

Interested persons are eligible for study participation if they are between 50-80 years of age, and have had histologically confirmed colorectal adenomas or serrated lesions removed during colonoscopy within the last 6 months. All participants of the study must be in good performance status at study entrance, corresponding to a level below 2 in the ECGG performance status scale [26]. Only patients able to give informed consent will be included after written informed consent.

Exclusion criteria for study participation are a history of hereditary nonpolyposis colorectal cancer (HNPCC) or familial adenomatous polyposis (FAP), a history of colorectal cancer or other concomitant cancers with the exception of basal cell carcinoma or curative treated cancers without actual anticancer medication. In addition, interested parties with intestinal malabsorption, short bowel syndrome or surgical bowel interventions leading to malabsorption will not be included into the study. Liver failure (hepatitis, cirrhosis), elevation of the liver enzymes ALT, AST or bilirubin to more than 2.5-fold above the upper normal value or inflammatory bowel disease are also exclusion criteria.

Regular intake of NSAIDs (including Cox-2 inhibitors) for more than 3 months per year, with the exception of low-dose ASS (≤ 100 mg per day), is not compatible with study participation. In addition, individuals who are on immunosuppressive medication are excluded from the study.

Individuals with impaired capacity to consent or who are impaired in swallowing pills will not be eligible for study participation.

In addition, subjects who have regularly consumed green tea extract with an EGCG content of more than 100 mg per day as a nutritional supplement for more than six months during the past two years will be excluded, as will individuals who have shown allergic reactions to green tea or its extract.

### Patient information and allocation

Informed consent is obtained after written and oral information by a study physician prior to inclusion into the study. The potential participants must fully understand all possible implications of the study, and all their questions must be answered sufficiently. After written informed consent, participants will be included into the one month run-in period where all participants receive 2 × 150 mg EGCG daily over 4 weeks. At study inclusion and after this period, liver enzymes (AST, ALT, bilirubin) will be checked and patients who either have more than 20 capsules (out of 64) left or have elevated liver enzymes of more than 2.5-fold of the upper normal value will be excluded from the study.

After the one month run-in period with ECGC, patients will be assessed and randomized to either the intervention group, which will receive green tea extract containing 300 mg EGCG per day, or to the control group, which will receive a placebo capsule twice daily.

### Blinding and randomization

Stratified randomization based on study centers and on regular intake of low-dose ASS (yes or no) will be performed with the software ROM [27] to achieve balance of these baseline covariates. There will be no further stratification, since it is assumed that with a sample size of n = 1203 per group for the ITT collective, covariates like green tea consumption, age, sex, family history of colon cancer, medication and lifestyle will be equally distributed in both study arms.

Recruitment will be competitive and concurrent in about 30 colonoscopy screening centers throughout Germany. It will be assumed that each study center will recruit at least 30 patients per year.

Patients and physicians including the study coordinators will be fully blinded to the study substance. An independent pharmaceutical contract manufacturer specializing in solid pharmaceutical dosing forms will produce the placebo and EGCG capsules, and will dispense them according to a randomization list. Patients included into the run-in phase will be notified by a fax to the study coordinating center.

Randomization will take place after a successful run-in period, and a random study number will be assigned to each participant. The study substance for the first three months (three packages containing 70 blistered capsules each) will then be sent out during one week after the end of the run-in period. Study start is the first day of intake of the study substance.

### Study procedures

#### Green tea extract

Powdered decaffeinated green tea extract will be prepared from the young unfermented leaf and leaf buds of Camellia Sinensis (L.) Kuntze (Fam. *Theaceae*) imported from China. The normally associated caffeine found in lower grade tea catechin mixtures will be eliminated to less than 0.5% content in the extract. The extract will be prepared using ethanol, methanol, acetone or water, as solvents. Microbiological and toxicological analyses are done to control for degradation products, pesticides, herbicides, polyaromatic hydrocarbons, and other unacceptable contaminants.

The study material will be packed in hard gelatine capsules containing either green tea extract (150 mg EGCG) or placebo, and pharmaceutical excipients consisting of microcrystalline cellulose, colloidal silicon dioxide, and magnesium stearate. The caffeine content of the green tea extract used to prepare the capsules will be maximally 0.5% and thus the caffeine content of one capsule will be below 1.5 mg.

#### Dose determination

The application of green tea extract standardized to a twice daily dose of 150 mg EGCG was decided upon a review of the existing literature on clinical long-term prevention studies and the rationale that a nutriprevention study should involve doses corresponding to nutritional use of green tea for which there is at least some epidemiological evidence for a cancer-preventive action.

A simple calculation shows that a daily dose of 300 mg EGCG corresponds to a normal intake of green tea: one cup of green tea is brewed with approximately 2 g of tea leaves. Tea leaves have a catechin content of about 10%. If the tea is brewed for 10 min, about 80% of the catechins will have dissolved. The amount of EGCG is about 50% of the catechins. This means that one cup of this green tea would contain about 80 mg EGCG, and the same amount of other dissolved catechins. Thus, 300 mg would correspond to 4 cups of tea brewed with this method. In practice, tea may be brewed with a shorter duration, which would result in a lower concentration of catechins. Thus, the daily dose applied in this study certainly lies within the range of 4-7 cups of tea per day, which is still a physiological dose.

A check with the literature reveals that in all existing long term studies, similar doses have been given (see table 1).

**Table 1:** summary of long term (≥ 2 weeks) interventional studies with green tea extract

Study	Daily intake of EGCG	Duration of trial	Sample size	Total catechins	EGCG	ECG	EGC	EC	Caffeine
[38]	300 mg	12 months	60	76%	52%	6%	6%	12%	0.1%
[7]	350 mg	2 months	69	84%	Not specified				19%
[31]	800 mg	1 month	8	98%	70%		13%	11%	
[9]	150 mg	12 months	71	20%	11%	2%	7%	0.2%	3%
[39]	225 mg	3 months	64	100%	35%	17%	< 1%	< 1%	7%
[40]	800 mg	1 month	26	80%-98%	50-75%	12%			2%
[6]	2000 mg	6 months	5	98%	70%		13%	11%	
[2]	300 mg	2 weeks	42	100%	100%				

#### Side effects and contraindications

There are no known common side effects of green tea extract. A systematic review performed in 2008 by the United States Pharmacopeia, case reports and animal pharmacology/toxicology studies from 1966 to 2007 showed no clear evidence for EGCG-related toxicity, specifically liver enzyme elevation in a common dose range of up to 800 mg [28]. A total of 216 case reports on green tea products were analyzed, including 34 reports concerning liver damage. Twenty-seven reports pertaining to liver damage were categorized as possible causality and seven as probable causality [28]. Consumption of concentrated green tea extract on an empty stomach is more likely to lead to adverse effects than consumption in the fed state, since up to five-fold higher plasma concentrations have been observed with empty stomach compared to the fed state [29-31]. In our study, liver enzyme parameters will be tested prior to enrollment, after the one month run-in period and after 4, 12, 20 and 36 months, respectively. An increase of liver enzymes by 2.5-fold above the upper normal value will lead to discontinuation of the study medication. Participants will be urged to take the capsules at breakfast and dinner. There are no known contraindications for the intake of green tea extract. Nevertheless, we will test for hepatotoxicity after one month of EGCG intake.

### Interactions

The potential of EGCG to induce drug-drug interactions by the inhibition of major human cytochrome P450 isoenzymes has been studied *in vitro *using isoform-selective probe substrates for the major human CYP450 isoforms involved in drug-metabolism (CYP1A2, 2C9, 2C19, 2D6 and 3A4) [32]. Based on these *in vitro *results, no significant interactions are expected in man for EGCG with concomitantly administered compounds mainly metabolized by CYP1A2, CY2C9, CYP2C19, CYP2D6 and CYP3A4. Interactions via inhibition of transporters such as P glycoprotein or Multidrug Resistance Protein (MRP) by EGCG is possible, but has not been reported so far.

#### Study visits

Every four months during the 36 months of the whole study, the participants will be asked to visit the gastroenterological centers to check for any adverse events and medication changes, and to confirm with the physician the regular intake of the study medication. Blood sampling for liver enzyme testing will be performed after run in, and at the 4, 12, 20 and 36 month visits, and at 4, 20 and 36 months for biomarker sampling.

Data on health status, medication, and complaints about the study substance will be collected and the study diary will be reviewed. After each clinical visit, the study medication for the next study period will be dispensed. In cases of non-compliance or any disruption longer than four months of intake of EGCG, the study investigators will decide whether the participant may continue in the study.

The final study visit will be for the control colonoscopy three years after the start of the study. If additional colonoscopies have been performed during the three year interval, the results of those will be documented as well. The results of the colonoscopy as well as the histological confirmation of potential metachronous colorectal adenomas will be documented, as well as clinical data on medication, health status, complaints and adherence to capsule intake.

Follow-up visits are planned for three and six years after the end of the study in order to assess the long term development of adenomas since we know that it may take years to see the effect of a preventive treatment. As an example, the effect of ASS chemoprevention on colon carcinoma was assessed 10 years after [33].

#### Drop-out criteria

In the following situations, no study enrollment after the run-in is foreseen:

• If the pill count after the run-in period contains more than 30 capsules (out of 70 capsules).

• If the liver enzyme parameters are elevated above 2.5-fold of the upper normal value.

• Study participation will be terminated in the following occasions:

• in the case of any severe adverse event impairing further participation in the study

• If the adherence to study medication is poor (if > 35% of the capsules were not taken during more than one study period)

• the regular intake of the capsules is not assured, for any reason

• it becomes evident after the enrollment that the inclusion criteria have not been fulfilled

• a continuous (longer than 3 months) intake of the following drugs has become necessary: Any NSAID including COX-2 selective NSAIDs and ASS in a dose higher than 100 mg

• the patients desires to exit the study

• The liver enzyme parameters are elevated more than 2.5-fold of the upper normal value.

It is intended to include the data of the follow-up colonoscopy in the subjects that have dropped out for the intention-to-treat analysis.

#### Biobanking

At time points prior to the run-in period, and after 4, 20 and 36 months, blood sampling for biobanking will be performed. The biobank will consist of pseudonymized (coded) plasma, serum and whole blood samples for biomarkers for adenoma development, as well as paraffin blocks of advanced adenomas detected in study participants at the 3 year colonoscopy. Participation in the clinical trial is independent from the biobanking project, and thus, separate informed consent will be given for the biobanking project.

### Ethics

The MIRACLE study will be performed according to the study protocol, as well as the ICH-GCP criteria, the Declaration of Helsinki, EU directives and applicable legal requirements. The study has been approved by Ethical Review Boards of the Martin Luther University of Halle-Wittenberg and Ulm University (Application 68/09, approved on the 9th of April 2009).

#### Data collection

Information on each patient's age, gender, height, weight, waist-to-hip ratio, ethnicity, regular tea intake, sport and activity level, smoking state, alcohol intake, family history of colon cancer and current disease and medication will be obtained from the initial interview before the run-in phase. From the colonoscopy prior to study entry, data on the number, localization and size of the removed adenoma(s) as well as the histological subtype and history of prior colonoscopies will be assessed. The patients' attitudes towards medicine and herbal supplements will be assessed at the beginning of the study.

At the follow-up visits, data on changes in concurrent disease or drug therapy, data on regular intake of the study medication and green tea beverages, laboratory data on liver enzymes and any adverse events experienced during the study will be recorded.

At the final visit, weight, waist-to-hip ratio, regular tea intake, data on changes in disease and medication will be obtained as well as data from the follow-up colonoscopy on the number, localization and size of the removed adenoma(s) as well as the histological subtype.

#### Data management

Data management, query management and monitoring of the study will be done by the Coordination Center for Clinical Trials at the Martin Luther University of Halle-Wittenberg (KKSH). Data collection will be done on paper CRFs and will be entered and stored in a validated GCP conform database. Study monitoring will be performed with one on-site visit per study center and regular data-entry checks of the CRFs.

#### Statistical analysis

The trial results will be analyzed by intention-to-treat and the per-protocol analysis. In analogy to [15] a modified intention-to-treat approach will be performed including all randomized subjects with a follow-up colonoscopy, irrespective of adherence to the study medication. Lacking values will be estimated using the Multiple Imputation MCMC method [34].

The per-protocol analysis will be done in all subjects who completed the whole course of the study. Safety will be assessed in all patients who took at least one capsule of the study medication.

##### Statistical analysis of the primary outcome

The study's aim is to check the following hypothesis: the regular intake of green tea extract (at least 300 mg daily) over three years results in a decrease of the incidences of colorectal adenomas. This primary outcome based on the intention-to-treat-population will be tested confirmatively by the one-sided χ^2^-Test [35] with a significance level of 5%.

The impact of other variables (e. g. study center, intake of low-dose ASS) related to the recurrence of adenomas will be explorative controlled by the logistic regression model [36]. Concerning the time of the recurrence of adenomas, the impact of other variables will be explorative controlled with the Cox proportional hazard model [37].

With the help of this multivariate analysis both the relative risks and the 95% confidence-intervals can be calculated.

##### Statistical analyses of the secondary outcome

How much other impact factors affect the risk of the incidence of metachronous adenomas will be analyzed explorative.

In addition, genetic and biochemical biomarkers for the recurrence of adenomas or the development of dysplasia and carcinoma will be analyzed explorative in the blood samples entered into the biobanking subproject.

#### Time plan

Patient recruitment is planned for a period of three years starting in October 2011. The total duration of the trial will be six years, with the last patient out in fall 2017.

## Discussion

The aim of this large prevention trial is to study the influence of regular nutritional supplementation with green tea extract containing EGCG, one of the active components of green tea, on the incidences of metachronous colorectal adenomas in subjects who have undergone polypectomy. Thus, it is a secondary prevention trial

The limitation of prior large-scale chemoprevention trials, when administering drugs like ASS or COX-2 inhibitors to a large group of healthy individuals for long term treatment, was safety. Even if the trials were successful with respect to their primary endpoint, prevention of metachronous colorectal adenomas, the clinical utility of NSAIDs used for this indication is rather low due to the unwanted side effects of these drugs.

This is the first large-scale controlled prevention trial using nutritional supplement as the active principle. The dose of ECGC in our green tea extract was chosen to mimic high and regular green tea consumption without the influence of caffeine. While we are aware that 300 mg ECGC per day is lower than the dose range in some trials (with multiple dose data of up to 800 mg per day), we chose this dose because it corresponds to a somewhat physiological intake of green tea of about 4-7cups per day, and because this dose is intended for use over an extended period of time, i.e. three years.

Of course, the mere act of taking a capsule two times a day might discourage some individuals from performing an individual preventive plan, but in principle this would be possible, and could be a recommendation for a broader target audience.

There are some data indicating that the other polyphenols in green tea may also contribute to its beneficial effects. Therefore we chose to use green tea extract containing a defined amount of ECGC but also the other polyphenols that are found in green tea.

We believe that a randomized controlled trial design is the only appropriate method to perform such a preventive trial, and justifies the efforts of such a trial. The limitations of the trial are the relatively short time period for follow-up (three years is short compared to the adenoma carcinoma sequence), the lack of a regular adherence measurement (ECGC does not accumulate and has a short half life, and therefore there is no long term parameter for the intake of EGCG), and the risk of a higher than presumed drop-out rate. However, the beneficial safety profile of green tea extract, and the widespread evidence on its cancer preventive potential, justifies in our view a validation of this compound for the nutriprevention of colorectal adenoma. Good accessibility and low costs might render this neutraceutical a top candidate for a wider use as food supplement in colon cancer prevention.

## List of abbreviations

ASS: Acetyl salicylic acid/; COX: Cyclooxygenase; CRF: Case report forms; CYP450: Cytochrome P450 enzyme; EC: Epicatechin; ECG: Epicatechin-3-gallate; EGC: Epigallocatechin; EGCG: Epigallocatechin gallate; FAP: Familial adenomatous polyposis; GCP: Good clinical practice; HNPCC: Hereditary nonpolyposis colorectal cancer; ICH: International conference of harmonization; MIRACLE: Minimizing the Risk of Metachronous Adenomas of the Colorectum with Green Tea Extract Study; MRP: Multidrug Resistance Protein; NSAID: Nonsteroidal anti-inflammatory drug.

## Competing interests

None of the authors has any competing interest (political, personal, religious, ideological, academic, intellectual, commercial or any other) to declare in relation to this manuscript.

## Authors' contributions

JS conceived of the study (together with TS) and drafted the manuscript. TE participated in the design and coordination of the study and helped to draft the manuscript. RM planned the sample size estimation, trial analysis and randomization and participated in the study design. MW participated in the statistical aspects of the study design and developed the randomization plan. JB prepared the nutrikinetic parts of the study, carried out the plasma analytics kinetic data analyses and participated in the design of the study. AS participated in the design and planning of the study. TS is the principal investigator and conceived of the study (together with JS) and drafted the manuscript. All authors read and approved the final manuscript.

## Pre-publication history

The pre-publication history for this paper can be accessed here:

http://www.biomedcentral.com/1471-2407/11/360/prepub
